# Molecular-Based Diversity Studies and Field Surveys Are Not Mutually Exclusive: On the Importance of Integrated Methodologies in Mycological Research

**DOI:** 10.3389/ffunb.2022.860777

**Published:** 2022-03-25

**Authors:** Jonathan Cazabonne, Lachlan Bartrop, Glen Dierickx, Yusufjon Gafforov, Tina A. Hofmann, Thomas E. Martin, Meike Piepenbring, Mauro Rivas-Ferreiro, Danny Haelewaters

**Affiliations:** ^1^Groupe de Recherche en Écologie de la MRC Abitibi (GREMA), Institut de Recherche sur les Forêts (IRF), Université du Québec en Abitibi-Témiscamingue, Amos, QC, Canada; ^2^Helles Brisbane, QLD, Australia; ^3^Research Group Mycology, Department of Biology, Ghent University, Ghent, Belgium; ^4^Research Institute for Nature and Forest (INBO), Brussels, Belgium; ^5^Laboratory of Mycology, Institute of Botany, Academy of Sciences of Uzbekistan, Tashkent, Uzbekistan; ^6^State Key Laboratory of Mycology, Institute of Microbiology, Chinese Academy of Sciences, Beijing, China; ^7^Senckenberg Biodiversity and Climate Research Institute (SBiK-F), Goethe-Universität Frankfurt am Main, Frankfurt am Main, Germany; ^8^Centro de Investigaciones Micológicas (CIMi), Herbario UCH, Universidad Autónoma de Chiriquí, David, Panama; ^9^Operation Wallacea Ltd, Wallace House, Old Bolingbroke, United Kingdom; ^10^Mycology Working Group, Goethe-Universität Frankfurt am Main, Frankfurt am Main, Germany; ^11^Population Genetics and Cytogenetics Group, Facultade de Bioloxía, Universidade de Vigo, Vigo, Spain; ^12^Faculty of Science, University of South Bohemia, Ceské Budějovice, Czechia

**Keywords:** fieldwork, taxonomy, monitoring, fungal diversity, fungal conservation, genetics

## Abstract

Understanding and describing the diversity of living organisms is a great challenge. Fungi have for a long time been, and unfortunately still are, underestimated when it comes to taxonomic research. The foundations were laid by the first mycologists through field observations. These important fundamental works have been and remain vital reference works. Nevertheless, a non-negligible part of the studied funga escaped their attention. Thanks to modern developments in molecular techniques, the study of fungal diversity has been revolutionized in terms of tools and knowledge. Despite a number of disadvantages inherent to these techniques, traditional field-based inventory work has been increasingly superseded and neglected. This perspective aims to demonstrate the central importance of field-based research in fungal diversity studies, and encourages researchers not to be blinded by the sole use of molecular methods.

## Introduction

  *We* must *collect, collect, and collect*.

—Richard P. Korf (Reinventing taxonomy, 2005)

When we want to understand ecosystems and how they work, fungi are a keystone group. They contribute to many important ecological processes, as nutrient recyclers, dominant decomposers, regulators of the natural environment, and mutualists (Dighton, 2003; Bardgett and van der Putten, 2014; Powell and Rillig, 2018), and are indispensable elements in the composition and functioning of ecosystems (Christensen, 1993).

Fungi are found in practically all ecosystems and their predominance facilitates interconnectivity with other groups of organisms, constituting a considerable diversity of associations (Hock, 2012). Most of these are poorly studied, such as certain endophytic associations with plants (Dastogeer and Wylie, 2017), and many mysteries remain to be discovered regarding particular associations, including insect–fungal (Vega and Blackwell, 2005; Haelewaters et al., 2021a), algal–fungal (Hawksworth, 1988), bacterial–fungal (Deveau et al., 2018), and bat–fungal associations (Cunha et al., 2020). Some of them result in tripartite (e.g., Cardoza et al., 2006; Afkhami and Stinchcombe, 2016; Haelewaters et al., 2018) or even quadripartite (e.g., Currie, 2000; Piepenbring, 2015) interactions. Many associations are yet to be discovered, since only 2–6% of the estimated 2.2–6 million species are formally described (Taylor et al., 2014; Hibbett et al., 2016; Hawksworth and Lücking, 2017).

Due to “the magnitude of fungal diversity” (*fide* Hawksworth, 2001) and the large number of existing threats facing them, fungi should be an important conservation priority (Moore et al., 2001; Heilmann-Clausen et al., 2014). Paradoxically, the fungal kingdom has only very recently received a slight interest in international or national programs for the protection and conservation of biodiversity (Velázquez et al., 2010; Sadiković and Kuštera, 2013), especially when it comes to little-studied morphological groups such as microfungi (Arnolds, 2001; Gonçalves et al., 2021). To date, only 0.0091 to 0.025% of the estimated fungal diversity have an assessed conservation status (IUCN, 2022). Growing awareness of the lack of consideration and equivalence toward the fungal kingdom has prompted the launch of a global initiative to make fungi one of the priorities within conservation and agricultural policy frameworks: the FF&F proposal, Fauna, Flora, and Funga (Kuhar et al., 2018).

These initiatives, combined with a renewed interest in fungi transcending the boundaries of the scientific community (Irga et al., 2018), have raised a broader awareness of the knowledge gap in worldwide fungal biodiversity. Studying fungal biodiversity introduces considerable challenges in terms of ecological, taxonomic, systematic, and phylogenetic knowledge, and of fungal protection, conservation, and enhancement (Hibbett et al., 2016). Indeed, knowing *what* one is protecting allows one to protect it better—an important challenge considering the complexity of fungal identification and ongoing conflicts regarding species concepts (Lücking et al., 2020).

Methods for studying fungal biodiversity have evolved dramatically since mycology emerged as a scientific discipline. From an initial focus on field inventories and macroscopic observations, mycologists progressed to examinations of cellular structures, made possible by the advent of light microscopy. Electron microscopy later allowed for the analysis of ultrastructural characteristics, and, in recent decades, DNA characteristics can be evaluated due to the development of molecular techniques such as high-throughput sequencing (Schmit and Lodge, 2005; Oberwinkler, 2018). With increasingly complex methods and ever-growing quantities of data, there has been a trend for mycologists to become more specialized in dealing with particular subfields such as cytology, physiology, species delimitation, phylogenetics, phylogenomics, comparative genomics, ecology, et cetera. As a result, traditional field inventories, morphological investigation for identification, and alpha taxonomy are frequently neglected in favor of molecular methods and sequence data (Drew, 2011; Ríos-Saldaña et al., 2018).

## The Molecular Era: New Insights in Fungal Diversity

Taxonomic investigations of fungi based on morphological features of sexual and/or asexual states (teleomorph, anamorph) were challenging up until the early 2000s, because anamorph–teleomorph connections were often difficult to assess and many morphological characteristics are prone to convergency or are indistinguishable (e.g., yeasts, conidiogenesis). Taxa were classified within frameworks that were useful for that time but not necessarily phylogenetically informative—as was later discovered (e.g., Taylor et al., 2000). For example, the red-pigmented yeasts were traditionally grouped into *Rhodotorula* and *Sporobolomyces*, two asexual genera that are highly polyphyletic in their original sense, with many species having been combined in other genera (Fell et al., 2000; Aime et al., 2006; Bauer et al., 2006; Haelewaters et al., 2021b). Cryptic (i.e., morphologically identical) species can only be differentiated by a molecular (DNA) approach (Lücking et al., 2014).

Important developments of molecular methods have allowed fundamental advances in many fields of mycology (Morange et al., 1998). DNA barcoding (Kress and Erickson, 2012) has emerged as a tool to help differentiate and identify fungal collections, and may reveal morphologically and ecologically unpredictable systematic relationships (e.g., Gómez-Zapata et al., 2021). It has become the norm in taxonomy and phylogenetic studies, and is also being used in other subfields such as ethnomycology (Teke et al., 2018) and paleomycology (Bernicchia et al., 2006). Open-access sequence databases such as NCBI GenBank (https://www.ncbi.nlm.nih.gov/genbank/) have allowed for quick and easy identifications of sequenced specimens based on multiple molecular markers. By using base-pair sequences of DNA as characteristics to discriminate species and individuals, aided by high throughput sequencing techniques (e.g., Edwards et al., 2006), molecular approaches have become a vital component of studies in fungal diversity (e.g., Anderson and Cairney, 2004; Dentinger et al., 2016; Gafforov et al., 2017, 2020; Haelewaters et al., 2018; Dirks and Russell, 2020), biology (Oliver and Schweizer, 1999; Osiewacz, 2002), evolution (Bruns et al., 1991; Berbee and Taylor, 2001, 2010; Fraser and Heitman, 2004; Spatafora et al., 2018; Butler et al., 2021), biogeography (Peay and Matheny, 2016; Wang et al., 2021), and ecology (Weiss et al., 2004; Peay et al., 2008; Peay, 2014; van der Linde et al., 2018).

Metagenomics is the analysis of the collective genomes of organisms in their natural environment independent of specimens (Nilsson et al., 2019). The use of environmental DNA (eDNA) has revealed new branches of the Fungal Tree of Life (Tedersoo et al., 2014; Khan et al., 2020) and presented evidence of cryptic fungi in many environments (Jones et al., 2011; Baeza et al., 2017; Léveillé-Bourret et al., 2021; Runnel et al., 2021). The use of eDNA in biodiversity studies has made it possible to obtain new understandings of hitherto hidden fungal diversity within any given ecosystem. Without new mass sequencing technologies, the discovery of fungal communities present in air (Rosa et al., 2020), snow (Penton et al., 2013; Rosa et al., 2020), aquatic ecosystems (Grossart et al., 2019), biotic substrates (e.g., plants, insects, animals), and even abiotic substrates (e.g., rocks Bjelland and Ekman, 2005; Egidi et al., 2014; Selbmann et al., 2014; Liu et al., 2021) would not have been possible.

In addition to being crucial to basic fungal research, molecular tools also contribute to applied fungal research, as in forest management (Glaeser and Lindner, 2011; Stewart et al., 2018; Hagge et al., 2019; Purahong et al., 2019; Tomao et al., 2020) or ecosystem restoration programs (Nuñez et al., 2021). Other questions have recently been re-evaluated thanks to molecular techniques, such as the impact of natural (Vargas-Gastélum et al., 2015; Pérez-Izquierdo et al., 2020) and anthropogenic (Borriello et al., 2012; Lienhard et al., 2014; Tomao et al., 2020; Kim et al., 2021) disturbances on fungal diversity. Through the detection of endangered species (Gordon and van Norman, 2015), molecular methods have also enabled a new appreciation of fungal conservation priorities (Geml et al., 2014).

There now is a common consensus that human activities and their predicted intensification in coming decades are severe enough to represent a sixth ‘Anthropocene' mass-extinction event (Barnosky et al., 2011; Ceballos et al., 2015; IPBES, 2019). Associated biodiversity declines will include the fungi specific to the affected habitats (Antonelli et al., 2020), hence the urgent need to identify these at-risk species swiftly, so actions can be taken to protect them.

## Limitations of Modern Methods

Despite recent improvements in molecular biology, important limitations must be taken into account. The identification of fungi based solely on sequence analysis presents great risks and can lead to false identification (Haelewaters et al., 2018; Hofstetter et al., 2019) and erroneous ecological conclusions (Wutkowska et al., 2019) including under- or overrepresentation of fungal groups in biodiversity surveys (George et al., 2019). Defining species boundaries accurately is crucial for diversity estimations as it determines whether organisms are members of the same phylogenetic group. Notwithstanding, many fungi are only known from metabarcoding studies (Wu et al., 2019) and cannot be accommodated within the current framework of fungal nomenclature due to lack of physical specimens or cultures that can serve as types (Ryberg and Nilsson, 2018). The valid publication of new names (*sensu* Turland et al., 2018) recommends specimen-based molecular methods. However, the cultivation approach favors rapidly growing fungi (e.g., *Penicillium*) and excludes biotrophic species (Zhang et al., 2010; Cordier et al., 2012). Methods for isolation can also skew diversity estimates, e.g., the choice of growing medium.

Indeed, the success of identifying species based on sequences available in GenBank greatly depends on the amount of available reference sequences (Nilsson et al., 2006). No or very few reference sequences are available for many rare and threatened fungal species as well as poorly known fungi in unexplored geographic areas (Piepenbring et al., 2018; Haelewaters et al., 2021c). This has direct consequences on our understanding of fungal diversity and phylogenetic relationships, as topologies are strongly influenced by taxon sampling. An increasing number of sequences not identified to species level or identified as “uncultured fungus” are present in online sequence databases; this, too, makes molecular-based identification problematic (Hibbett et al., 2011; Nilsson et al., 2014). Nevertheless, efforts have been made to maximize the probability of accurate sequence-based identification, e.g., the UNITE database (Abarenkov et al., 2010) and the use of the term “species hypothesis”—a cluster of sequences that share 97–99% similarity (Kõljalg et al., 2013). However, despite these advances, the greatest challenge in fungal identification and barcoding will mostly, but not only, remain of a taxonomic nature (Nilsson et al., 2006).

The success and efficacy of molecular protocols, including DNA extraction, PCR amplification, and sequencing can be questionable, which may lead to partial or poor-quality sequences. DNA extraction remains difficult for certain fungal groups, requiring specialized protocols or modifications of standard kits (Haelewaters et al., 2015; Sundberg et al., 2018; Meswaet et al., 2021). PCR amplification may depend on taxon-specific primers to target universal or secondary (meta-) barcoding markers (Liu et al., 2020; Reynolds et al., 2021) that are selective for certain taxa. In fungal groups where the internal transcribed spacer (ITS) region of the ribosomal RNA gene does not provide sufficient resolution, secondary DNA barcodes need to be used, representing additional inputs of time and cost (Lücking et al., 2020).

In addition to the pitfalls in sequence-based identification and current public sequence databases, homopolymer regions are also problematic. In addition to practical and technical issues, the use of molecular methods may lead to statistical problems, such as the question of the normalization of fungal taxa abundance among samples (McMurdie and Holmes, 2014; Weiss et al., 2017; Lin and Peddada, 2020).

Lack of funding and training is also a major concern. Molecular work is expensive and highly specialized, and requires specific equipment not available for many mycologists (Hibbett et al., 2016). This is a problem especially in remote and understudied areas in the tropics (Haelewaters et al., 2021a,c) and some temperate zones, such as Central Asia (Gafforov, 2017; Cheek et al., 2020; Abdurazakov et al., 2021).

## Field-Based Research: The Solution?

Field surveys are one of the oldest and most fundamental techniques for studying biodiversity, through the observation and description of biological organisms living on Earth. Unfortunately, their value as viewed by the scientific community has diminished and today species records are rarely a priority for researchers. A decline in the relative importance of field-based science has been observed in most taxa (Ríos-Saldaña et al., 2018). This may be particularly acute in mycology, perhaps due to the cryptic nature of fungi, resulting in tedious and costly species identifications that require expertise from mycologists, taxonomists, and evolutionary biologists. This is a worrying trend, given field surveys generate essential new information that cannot be obtained through the prism of sequencing, and are ideal instruments for training the next generation.

### Taxonomy

Alpha-taxonomy (i.e., taxonomy in its historical sense) establishes taxonomic units based on phenotypic traits (Simpson, 1960). Traditionally, this was achieved mainly by the presentation of monographs and allowed for the study of the variability of informative characteristics. Diagnosable morphological features can be attained rapidly in the field as well as by light microscopy in the lab, and multiple specimens of species warrant insights into morphological variation, which forms the basis of species delimitation (Noordeloos and Antonín, 2008; Kerekes and Desjardin, 2009; Phengsintham et al., 2013). Some characteristics important for the morphological identification of species can only be observed on freshly collected specimens, including the original color, taste, smell, chemical reactions, e.g., in the case of fruiting bodies of *Russula* spp. (Adamčík et al., 2019), and the organization of oil drops in ascospores (Baral, 1992). Field survey methods (Mueller et al., 2004) allow access to macro- and micro-morphology of a given taxon that is combined with previously or newly generated sequences. This “fusion” between phenotypic and molecular genetic data, referred to as integrative taxonomy (Dayrat, 2005; Pante et al., 2015), is essential for species delimitation and, thus, formal description of taxa new to science (Simões et al., 2013).

### Geographic Records of Known Species

Despite a generalized decline in the importance of traditional taxonomic research (Drew, 2011), new records of known species should be properly documented and published. To establish a species concept new to science is easy, but it is more valuable to apply an old name to a new collection, although this takes more time and effort. As a result, the new information is connected to existing data. With every new collection, the knowledge of any given species is improved; this includes infraspecific variation of morphological and molecular characteristics, interaction with other organisms (host range in the case of pathogens), and geographic distribution. A high number of geographic records are required to become aware of changes in distributional areas and general patterns of spatial distribution, which is critical in the light of climate change and for species that are disease agents of humans, animals, or cultivated plants. Good knowledge of geographic distributions is indispensable for phylogeographic studies and forms the basis of assessments for the International Union for Conservation of Nature (IUCN) Red List of Threatened Species and designation of Alliance for Zero Extinction (AZE) sites (https://zeroextinction.org/).

### Ecology

Opportunistic field sampling, the most common approach used by citizen scientists (Mueller, 2017), can be of great value with regard to fungal studies, particularly when exploring poorly-studied and remote locations (Rivas-Ferreiro, 2021). Long-term field monitoring, however, has the potential to generate longitudinal datasets necessary to understand the role of fungi in ecosystems. Not only do data from such monitoring studies enable hypothesis-driven research (Korf, 2005), they inform on fungal reproduction (i.e., sexual states), phenology (e.g., Jumbam et al., 2019), life cycles (i.e., methods of spore dispersal), nourishment (i.e., saprophytic, endophytic, etc.), and functional diversity. Ecological monitoring also reveals key information about ecological adaptations, i.e., insects attracted by volatiles (Davis and Landolt, 2013), nematode-trapping structures (Zhang et al., 2013), secotioid basidiomata as adaptations to dry environments (Claridge et al., 2000a,b), the dominance of sporocarps in periods with high precipitation (e.g., López-Quintero et al., 2012; Piepenbring et al., 2015), et cetera.

### Traits

In addition to capturing ecological information as outlined above, observations made in the field allow to integrate biotic and abiotic data, resulting in information critical for fungal conservation studies. This includes ecological observations concerning the relevance of, e.g., climate (temperate, tropical), seasonality, weather, environmental factors (pH, water, nutrient availability, substrate, soil characteristics), and habitat, as well as insights concerning dispersal strategies, interactions with other organisms (natural enemies, hosts), and responses to anthropogenic pressures. These data also facilitate the discovery of abiotic and biotic connections between fungal clades and biodiversity more broadly (Kendrick, 2011; Peay et al., 2016). Meta-analysis and ecological studies rely on key spatial data obtained from surveys (Shrestha and Bawa, 2014; Yan et al., 2017) and temporal variations of fungal diversity governed by natural and anthropogenic environmental change can advise conservation efforts (Haelewaters et al., 2021c; Kaishian, 2021).

### Cultures

Field surveys allow the isolation of fungal collections from habitats such as plant leaves, fruit skins, and insect guts that are common sources of endophytes, yeasts, and yeast-like fungi, often representing untapped diversity preserved in a metabolically inactive state (Boundy-Mills et al., 2015; Sharma et al., 2019). From here, results obtained by molecular methods can be supplemented with morphological characteristics (including photographs of colonies on distinct culture media; Pitt et al., 1983), spectral analyses (Petisco et al., 2008; Rodrigues et al., 2011), recognition of anamorphic and teleomorphic states (Hawksworth, 2011), and screening for biochemical profiles (Suh et al., 2008).

### Fungarium Collections

Specimens collected through field surveys are often deposited in fungaria, after having been slowly dried, packaged, and labeled. Fungarium collections represent a treasure trove of data because they represent traces of past and current species diversity and occurrences (Paton et al., 2020). Even if unidentified, fungarium collections may be useful for future researchers who may investigate morphological or molecular characteristics (Brock et al., 2009; Osmundson et al., 2013; Dentinger et al., 2016). Specimens from field surveys, as a best practice, are accompanied by field notes with information about characteristics that are only present *in situ* (e.g., hygrophany, smell, substrate) or that are no longer observable after drying (e.g., chemical reactions, colors, texture).

### Checklists

The value of checklists resulting from field surveys is increasingly appreciated by many researchers worldwide (Piepenbring et al., 2011, 2020; Haelewaters et al., 2021c). A checklist of fungi for a given area presents information about the history of mycological research in that area, generates data necessary to compare the area with other regions with respect to species diversity, helps to detect and monitor invasive species, and advances taxonomic research with new collections of “forgotten” species or “old names” (Piepenbring et al., 2020). Some researchers have made it a matter of principle to publish fungal species checklists (e.g., Marinho et al., 2018; Diederich and Ertz, 2020; Fryar et al., 2020). This helps fill the knowledge gaps on fungal diversity, particularly in under-sampled regions. Recent funded initiatives have highlighted the critical value of publishing checklists supported with molecular data (e.g., Haelewaters et al., 2018; Gafforov et al., 2020); we highly encourage our mycological colleagues to do the same. Fungal species occurrence data, whether from protected areas or public lands, through amateur or grant-funded, scientific surveys, are critical to science.

### Training

Field-based projects can and should bring together researchers spanning junior to senior scientists as well as students and interns from diverse backgrounds. Teaching in the field is an effective way to educate young mycologists. Collaborators in the area may identify interested and talented students who will participate in every part of the research, in addition to local people to be trained as parataxonomists. Such education should go further than collecting fungi; it may include seminar talks by senior participants, presentations by more experienced students, interactions in the field, and laboratory work (Piepenbring and Yorou, 2017).

## Unity Makes Strength: Onward Together!

Fungal diversity can only be unraveled and interpreted by combining molecular and organismic approaches that take into account the complexity and wholeness of members of the kingdom. In the field, only a small part of the existing fungal diversity can be seen through above- or belowground spore-bearing structures that are easily observed. A bias exists toward well-known species of “macrofungi” (Gonçalves et al., 2021). Indeed, “microfungi”—e.g., endophytes, epiphytes, arbuscular mycorrhizal fungi, coprophilous fungi—are neglected in fungal diversity and conservation studies. Most fungal diversity resides underground and in living or dead organisms, by means of hyphal networks, most of them unable to produce sporocarps (Halme et al., 2012). The vast majority of fungal taxa studies are only possible by combining molecular and field survey methods to an integrative double-sided focus.

In order to know the funga worldwide, we need field survey projects, particularly in poorly studied areas. Funding programs focusing on fieldwork, capacity building (to provide access to molecular equipment, especially in the tropics and the Global South), and taxonomy are key. An example is the U.S. National Science Foundation Poorly Sampled and Unknown Taxa (PurSUiT) category. In addition, grants should include funds to hire specialized taxonomists to accelerate species identifications. In remote areas lacking mycologists, such as in most of the tropics, Indigenous people may possess ancestral knowledge on fungi. To go further, we must encourage the establishment of field surveys by actively soliciting contributions of local communities. Such integrative parataxonomy can be of great value in terms of knowledge and scientific popularization. In the same vein, integrative taxonomy is directly linked to the use of this powerful combined method. Taxonomy should be based on a combination of molecular phylogenetic, morphological, and ecological data. Fungal biodiversity or ecological studies based on molecular data only, or equally without such molecular sequence data, can be considered as partly “blind.”

In every fungal diversity study, the collect-and-sequence approach (Truong et al., 2017) should be employed as standard. Where these two main methods are employed together, new perspectives can be explored. For example, eDNA and field surveys can be complementary tools when it comes to studying fungal communities and forest management (Yan et al., 2018). Cryptic fungi (e.g., soil fungi, epiphytes, endophytes) that cannot (or are very difficult to) collect or culture on axenic media can be molecularly identified and associated with ecological information (seasonality, spatial distribution) through employing an integrated methodology. We do note that for a few ecosystems, molecular approaches remain our best bet. For example, desert ecosystems, Antarctica, deep sea sediments, and other extreme ecosystems, where sporocarps are never seen and for which culturing techniques are very challenging (i.e., culturing of fungi under high pressure, or under very specific nutrient availability). Likewise, some taxa that we have failed to find in metabarcoding studies and that are unculturable but that can be readily observed and collected in the field, may continue to benefit from “traditional” taxonomic studies.

Integrative methodologies are crucial for improving our understanding and knowledge of global fungal diversity, both from a fundamental and applied research perspective. Indeed, integrative approaches are poised to become even more crucial for fungal conservation as new initiatives arise to protect fungi (Kuhar et al., 2018) and to consider all fungal species in biodiversity goals (Gonçalves et al., 2021). Only a tiny fraction of the total fungal biodiversity on the planet is known, and there is a huge need (i) to improve our understanding of the fundamental role of fungi in the functioning of ecosystems, (ii) to characterize the patterns of the distribution of biodiversity and the dynamics of fungal communities, whether within endangered habitats or not, (iii) to improve taxonomic knowledge, and (iv) to fully integrate the fungi into global conservation networks, such as the IUCN Red List, AZE sites, and Key Biodiversity Area (KBD) designations. Before it is too late.

## Data Availability Statement

The original contributions presented in the study are included in the article/supplementary material, further inquiries can be directed to the corresponding author/s.

## Author Contributions

JC and DH: conceptualization and writing—original draft and visualization. JC, LB, GD, YG, TAH, TEM, MP, MR-F, and DH: writing—review and editing. All authors have read and agreed to the published version of the manuscript. All authors approved the submitted version.

## Funding

This work was supported in part by the Research Foundation—Flanders (Fellowship Fundamental Research 1126221N to GD, financed by the Research Institute for Nature and Forest; Junior Postdoctoral Fellowship 1206620N to DH), Panama's Secretaría Nacional de Ciencia, Tecnología e Innovación (SENACYT, grant number 121-2017-4-EIE17-016 to TAH), the German Federal Ministry of Education and Research (BMBF) (grant number 01DG20015FunTrAf to MP), the Spanish Ministry of Education (Predoctoral Fellowship FPU20/02280 to MR-F), and the U.S. National Science Foundation (DEB-2018098 and DEB-2127290 to DH).

## Conflict of Interest

TEM and DH were employed by Operation Wallacea Ltd. The remaining authors declare that the research was conducted in the absence of any commercial or financial relationships that could be construed as a potential conflict of interest.

## Publisher's Note

All claims expressed in this article are solely those of the authors and do not necessarily represent those of their affiliated organizations, or those of the publisher, the editors and the reviewers. Any product that may be evaluated in this article, or claim that may be made by its manufacturer, is not guaranteed or endorsed by the publisher.
